# A brain specific alternatively spliced isoform of nonmuscle myosin IIA lacks its mechanoenzymatic activities

**DOI:** 10.1016/j.jbc.2023.105143

**Published:** 2023-08-09

**Authors:** Samprita Das, Ditipriya Mallick, Sourav Sarkar, Neil Billington, James R. Sellers, Siddhartha S. Jana

**Affiliations:** 1 School of Biological Sciences, Indian Association for the Cultivation of Science, Kolkata, West Bengal, India; 2 Laboratory of Molecular Physiology, National Heart, Lung and Blood Institute, National Institutes of Health, Bethesda, Maryland, USA

**Keywords:** nonmuscle myosin II, alternative splicing, myosin filament formation, actin-gliding activity, enzymatic Mg^2+^-ATPase activity, circadian rhythm

## Abstract

Recent genomic studies reported that 90 to 95% of human genes can undergo alternative splicing, by which multiple isoforms of proteins are synthesized. However, the functional consequences of most of the isoforms are largely unknown. Here, we report a novel alternatively spliced isoform of nonmuscle myosin IIA (NM IIA), called NM IIA2, which is generated by the inclusion of 21 amino acids near the actin-binding region (loop 2) of the head domain of heavy chains. Expression of NM IIA2 is found exclusively in the brain tissue, where it reaches a maximum level at 24 h during the circadian rhythm. The actin-dependent Mg^2+^-ATPase activity and *in vitro* motility assays reveal that NM IIA2 lacks its motor activities but localizes with actin filaments in cells. Interestingly, NM IIA2 can also make heterofilaments with NM IIA0 (noninserted isoform of NM IIA) and can retard the *in vitro* motility of NM IIA, when the two are mixed. Altogether, our findings provide the functional importance of a previously unknown alternatively spliced isoform, NM IIA2, and its potential physiological role in regulating NM IIA activity in the brain.

Alternative splicing is a key posttranscriptional process to create diverse transcripts and protein isoforms from a single gene for the generation of more than 90,000 proteins from ∼25,000 human genes (1, 2). In this process, some exons from a transcribed pre-mRNA are skipped or retained allowing the generation of a number of unique mature mRNAs. This process is highly specific to tissue, cell type, and various stages during development. The spatiotemporal effect of the alternative splicing events of each gene during development and the biochemical relevance among the isoforms synthesized from a single gene are still under investigation (3, 4, 5).

Alternative splicing has been established in genes encoding class II family of conventional myosin motor proteins, nonmuscle myosin II (NM II) (6, 7, 8, 9). Typically, NM II molecules contain a pair of each of the heavy chains (HC), regulatory light chains (RLC), and essential light chains. Vertebrates have three NM II paralogs, based on their HCs, namely nonmuscle myosin heavy chain (NMHC) IIA, NMHC IIB, and NMHC IIC, which are encoded by three different genes, *MYH9*, *MYH10*, and *MYH14*, respectively (10, 11). NM II molecules are characterized by their distinct motor activities, actin-activated Mg^2+^-ATPase activities, and bipolar filament formation. The bipolar filaments are essential in a variety of cellular functions like cell movement, division, and cytoskeletal organization (12).

Previous reports have shown the occurrence of alternative splicing at loop 1 (near the ATP-binding region) and loop 2 (near the actin-binding region) positions of motor domain of NMHC IIB and NMHC IIC (6, 7, 8, 9). NM IIB with insertion at loop 1 of HC is designated NM IIB1, insertion at loop 2 is designated NM IIB2, insertion at both loops is NM IIB12. The non-inserted variant is designated NM IIB0 (or simply NM IIB). This nomenclature format is also retained for other NM IIs. Insertion of 10 or 16 aa at the loop 1 of NMHC IIB (NM IIB1a or -B1b, respectively) is neuronal tissue-specific and it makes up most of the NMHC IIB mRNA in the human cerebrum and retina (13). The expression of these isoforms correlates with neuronal cell differentiation, facial neuron migration, and inhibition of cell division (13, 14). The 10 aa inserted isoform, NM IIB1a, shows only a small increase in the Mg^2+^-ATPase activity and the *in vitro* motility compared to the non-inserted isoform (15). In contrast, the insertion of 8 aa at loop 1 of NMHC IIC is detectable in a variety of tissues, including liver, kidney, testes, brain, lung and it shows a higher rate of both *in vitro* motility and actin-activated Mg^2+^-ATPase activity (8). On the other hand, insertion of 21 aa or 33 aa at loop 2 of NMHC IIB and IIC, respectively, is neuronal tissue specific (9, 16). Kim *et al.* (16) concluded that NM IIB2 (21 aa) lacks enzymatic activity but plays an important role in cerebellar development, such as, Purkinje cell localization and maturation, dendrites and the dendritic spine formation (14). A further study using full length protein concluded that although the IIB2 has an order of magnitude lower actin activated ATPase than the non-inserted variant, it nonetheless has a measurable activity (11). Deletion of this isoform causes a reduced number of spines and dendrite branches associated with Purkinje cells, which cannot be rescued by any other spliced isoform. The aa sequence of loop 2 insert of NMHC IIC is 67% identical between humans and mice, with the complete identity of aa in the flanking regions. Interestingly, it has been established that NM IIC2’s activity is not dependent on phosphorylation and that NM IIC1C2 behaves the same. Interestingly, the composition of NM IIC changes from phosphorylation-dependent NM IIC1 to phosphorylation independent NM IIC1C2 at both mRNA and protein levels during differentiation, indicating how the splicing event can regulate NM IIC activity during the differentiation process (17). In contrast to these findings, no such alternative splicing event for NMHC IIA has been reported to date.

In this study, we discover an insertion of 21 aa (63 nt) due to alternative splicing at the loop 2 region of NMHC IIA, which is specific to the brain tissue. Using the baculovirus expression system we provide evidence that the loop 2 inserted isoform, NM IIA2 has low actin-activated ATPase activity and lacks measurable actin-gliding activity *in vitro*. We further find that the NMHC IIA2 isoform has a dominant reductive effect over the NM IIA0 in the actin-gliding activity *in vitro*. Interestingly, we observe a significant change in the expression of NMHC IIA2 with the circadian rhythm, suggesting a potential role of NMHC IIA2 in the day-night cycle.

## Results

### Identification of alternatively spliced A2 insert in NMHC IIA

**Figure 1:**
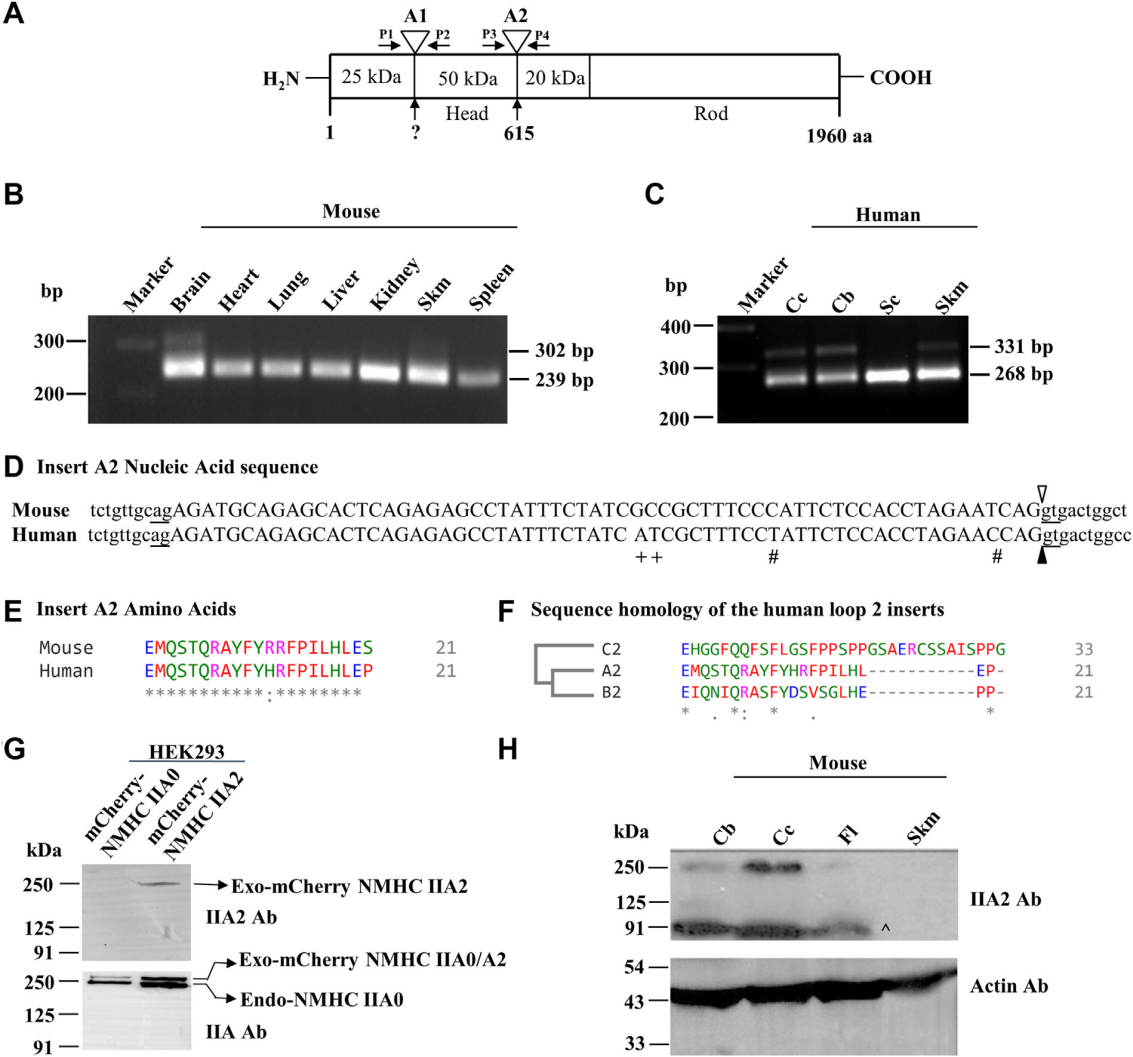
**Identification of A2 insert of NMHC IIA in mouse and human brain tissues.***A*, schematic diagram shows the possible splice sites at loop 1 (A1) and loop 2 (A2) of NMHC IIA, and the positions of primers (*arrows*). *B* and *C*, RT-PCR analysis of total RNA isolated from various mouse (*B*) and human (*C*) tissues, as indicated. Bands at 302 and 331 bp indicate inclusion of A2 exon in mouse and human tissues, respectively. *D*, nucleotide sequence alignment of mouse and human A2 exon (*uppercase*) and its flanking introns (*lowercase*). Note that the splice sites are indicated by *underlined* ag and gt nucleotides, and the exon A2 starts with the second nucleotide of the codon for glutamic acid, GAG. #, denotes the mismatch nucleotide which causes change in aa in mouse, + denotes the mismatch nucleotide with no aa change in A2 insert. *E* and *F*, amino acid sequence alignment of mouse and human A2 inserts (*E*), and human A2, C2, and B2 inserts (*F*) using Clustal Omega. Note that the aa sequence homology between mouse and human A2 inserts is 85.71%. aa, amino acids; ∗, conserved aa; :, strongly similar aa substitutions; ., weakly similar aa substitutions. *G*, immunoblot analyses of HEK293 cells expressing mCherry-tagged NMHC IIA0 or -IIA2 using antibodies against A2 insert (IIA2 Ab), and a C-terminal region (IIA Ab) that can detect both NMHC IIA0 and -IIA2. The exogenous mCherry-tagged NMHC IIA0 or -IIA2 moves slowly than the endogenous NMHC IIA0. *H*, immunoblot analyses of various regions of mouse brain and skeletal muscle using antibody against mouse A2 insert (IIA2 Ab). A nonspecific band near 91 kDa has appeared with this antibody, shown with “∧”, but not in the cell lysate (*G*). Actin was used as loading control. Data are representative of three independent experiments. Cb, cerebellum; Cc, cerebral cortex; Fl, frontal lobe; NMHC, nonmuscle myosin heavy chain; RT-PCR, reverse transcription; Sc, spinal cord; Skm, skeletal muscle.

Previous studies reported the existence of alternatively spliced inserts at loop 1 and loop 2 regions in the motor domains of NMHC IIB and IIC (6, 7, 8, 9), but not in NMHC IIA. We were interested to check if alternative splicing occurs at loop 1 and loop 2 regions of NMHC IIA mRNA using a modified reverse transcription-PCR (Fig. 1*A*). We screened a variety of mouse tissues using the primers flanking the putative splice sites in NMHC IIA, which are located at homologous locations to those found in NMHC IIB and IIC. We could not detect alternative splicing at loop 1 in NMHC IIA (Fig. S1) but, found that splicing was detectable at loop 2 in brain and skeletal muscle tissues (Fig. 1*B*). Bands with slower migration indicate the incorporation of a spliced exon (named A2) in NMHC IIA mRNA. Unlike NMHC IIB or IIC, the abundance of alternative splicing events at loop 2 of NMHC IIA was as low as 15% and 5% compared to that of the unspliced myosin in mouse brain and muscle, respectively. The exon A2 was also detectable in the human brain and skeletal muscle (Fig. 1*C*), suggesting that alternative splicing at loop 2 of NMHC IIA pre-mRNA is conserved among mammals. We performed sequence analysis of the slower migratory band from brain tissues and found that the 63 nt exon A2 shares 93.6% similarity with mouse and human (Fig. 1*D*). The expression of NMHC IIA2 mRNA in humans was further verified in the Gene Expression Omnibus database, and notably the expression of A2 exon in human was restricted to brain and skeletal muscle tissues (Fig. S1*B*). Similar to the insert B2, insert A2 exhibits >90% identity at the amino acid level between mouse and human (Fig. 1*E*). Phylogenetic analysis of amino acid sequences of spliced inserts at loop 2 suggests that A2 and B2 are more closely related to each other than to C2 (29% and 48% similarity, respectively) (Fig. 1*F*) in human, a finding similar to the overall identity of motor domain of their corresponding HCs (18).

To check if NMHC IIA2 mRNA can be expressed at the protein level, we first generated a polyclonal antibody against the mouse A2 insert in rabbit. We performed immunoblot analyses using the A2 insert specific antibody (A2 Ab) with HEK293 cell lysates, expressing exogenous mCherry-NMHC IIA0 or -NMHC IIA2. Figure 1*G* (*upper panel*) shows a band near 250 kDa with mCherry-NMHC IIA2 transfected cell lysate, but not with mCherry-NMHC IIA0, demonstrating that the antibody does not cross-react with the non-inserted isoform. Note that both endogenous NMHC IIA0 and exogenous mCherry-NMHC IIA0 or -IIA2 were detectable by an antibody (IIA Ab, *lower panel*), which is specific to the C-terminal region of NMHC IIA. Using the A2 specific antibody, we checked the expression of NMHC IIA2 in various regions of mouse brain tissue and noted that its expression in cerebral cortex, as indicated by the band near 250 kDa, was elevated compared to cerebellum or frontal lobe, suggesting the region-specific alternative splicing of NMHC IIA pre-mRNA (Fig. 1*H*). We could not detect NMHC IIA2 in the skeletal muscle although NMHC IIA2 mRNA was detectable, indicating that the abundance of NM IIA2 protein in the skeletal muscle was below the detection limit. In addition to the band near 250 kDa, a 90 kDa band was detected in the tissue lysate, but was absent in the immunoblot of HEK293. This indicates either the antibody can detect nonspecifically a protein in the tissue lysate or that proteolysis produces a fragment of myosin containing the motor domain. Altogether, these data suggest that NMHC IIA pre-mRNA undergoes alternative splicing at loop 2 and that the spliced exon is expressed at the protein level in brain tissue.

### NM IIA2 has very low actin-activated Mg^2+^-ATPase activity

**Figure 2:**
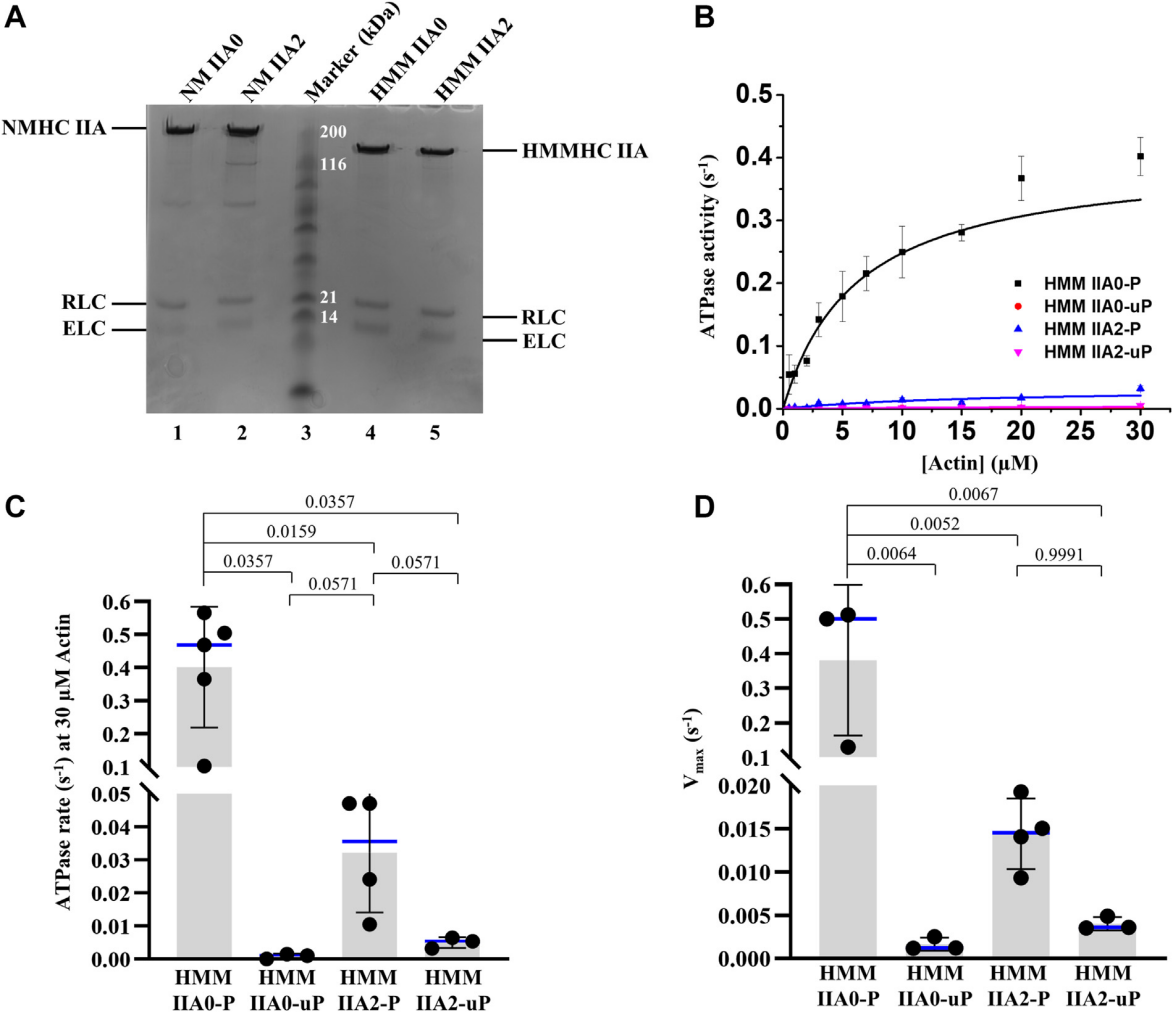
**HMM IIA2 does not show actin-activated Mg**^**2+**^**-ATPase activity.***A*, Coomassie blue staining of baculovirus expressed HMM IIA0, -IIA2, and full-length NM IIA0 and -IIA2. Heavy chain, essential light chain, and regulatory light chain are denoted as HC, ELC, and RLC, respectively. NM IIA0 (*lane 1*), NM IIA2 (*lane 2*), marker (*lane 3*), HMM IIA0 (*lane 4*), and HMM IIA2 (*lane 5*). *B*, the actin-activated Mg^2+^-ATPase activity of HMM IIA0 and -IIA2 in the presence (P) or absence (uP) of MLCK at varying concentration of actin (0–30 μM) at 25 °C. The similar behavior of phospho-HMM IIA2 with unphospho-HMM IIA0 indicates the enzymatic inactivity of the HMM IIA2 motor. *C*, quantification of *V*_max._ The data from (*B*) were fitted into the Michaelis–Menten equation and the kinetic constant *V*_max_ was measured. *D*, quantification of ATPase activity at 30 μM actin. Data are shown as mean ± SD from minimum of three experiments from three biological replicates. *Blue horizontal line* denotes the median value. Data were analyzed using the Kruskal–Wallis test followed by the pairwise Mann–Whitney U test. *p* > 0.05 was considered to be nonsignificant. HMM, heavy meromyosin; MLCK, myosin light chain kinase; NM, nonmuscle myosin.

To assess the biochemical properties of A2 insert containing isoform, NM IIA2, we used baculovirus/Sf9 expression system to produce purified full length myosin protein (NM IIA) and a heavy meromyosin-like (HMM IIA) fragment that is missing the filament forming tail region of the molecule. Figure 2*A* shows the purified NM IIA0 (without A2 insert) and IIA2 (with A2 insert) (*lanes 1* and *2*, respectively), and HMM IIA0 and IIA2 (*lanes 4* and *5*, respectively) molecules. Note that both inserted (IIA2) and non-inserted (IIA0) NM IIA molecules are composed of their respective HCs, RLCs and essential light chains. To evaluate the effect of A2 insert in the actin-activated ATPase activity, we measured the ATPase activity of HMM IIA2 at 0 to 30 μM actin in the presence or absence of RLC phosphorylation (Table S1). We considered noninserted HMM IIA0 as control in the assay, though experiments with HMM IIA0 have been reported elsewhere (19). Figure 2*B* demonstrates that the phosphorylated HMM IIA0 (HMM IIA0-P, black square) exhibited a large increase in Mg^2+^-ATPase activity with increasing concentration of actin, whereas the unphosphorylated HMM IIA0 (HMM IIA0-uP, red circle) showed no such increase in Mg^2+^-ATPase activity, a similar observation as previously reported (*V*_max_ for HMM IIA0-uP; 0.0016 ± 0.0008 s^−1^ and for HMM IIA0-P; 0.3809 ± 0.22 s^−1^) (19). On the contrary, phosphorylation of HMM IIA2 increased the maximal Mg^2+^-ATPase activities ∼4 fold only in the presence of actin (*V*_max_ for HMM IIA2-P, 0.0173 ± 0.004 s^−1^ and for HMM IIA2-uP, 0.0043 ± 0.0008 s^−1^, Fig. 2*C*). Interestingly, both HMM IIA2-uP and -IIA2-P had similar actin binding affinities as HMM IIA0-uP (Fig. 2*B*). We compared the ATPase activity of HMM IIA2 with HMM IIA0 at 30 μM actin and found that HMM IIA2 activity remained low even after phosphorylation (0.0321 ± 0.018 s^−1^), whereas phosphorylated HMM IIA0 showed 0.4018 ± 0.182 s^−1^ (Fig. 2*D*). The extent of phosphorylation of RLC by myosin light chain kinase (MLCK) was determined by urea gel electrophoresis. Fig. S2 indicates a faster migration of RLC in the presence of MLCK compared with the absence of MLCK, confirming the phosphorylation of RLC in the presence of MLCK in both HMM IIA0 and -IIA2 proteins. Altogether these data suggest that incorporation of A2 insert results in NM IIA that has a dramatically lowered activity relative to the noninserted NM IIA0.

### NM IIA2 inhibits *in vitro* motility activity of NM IIA0

**Figure 3:**
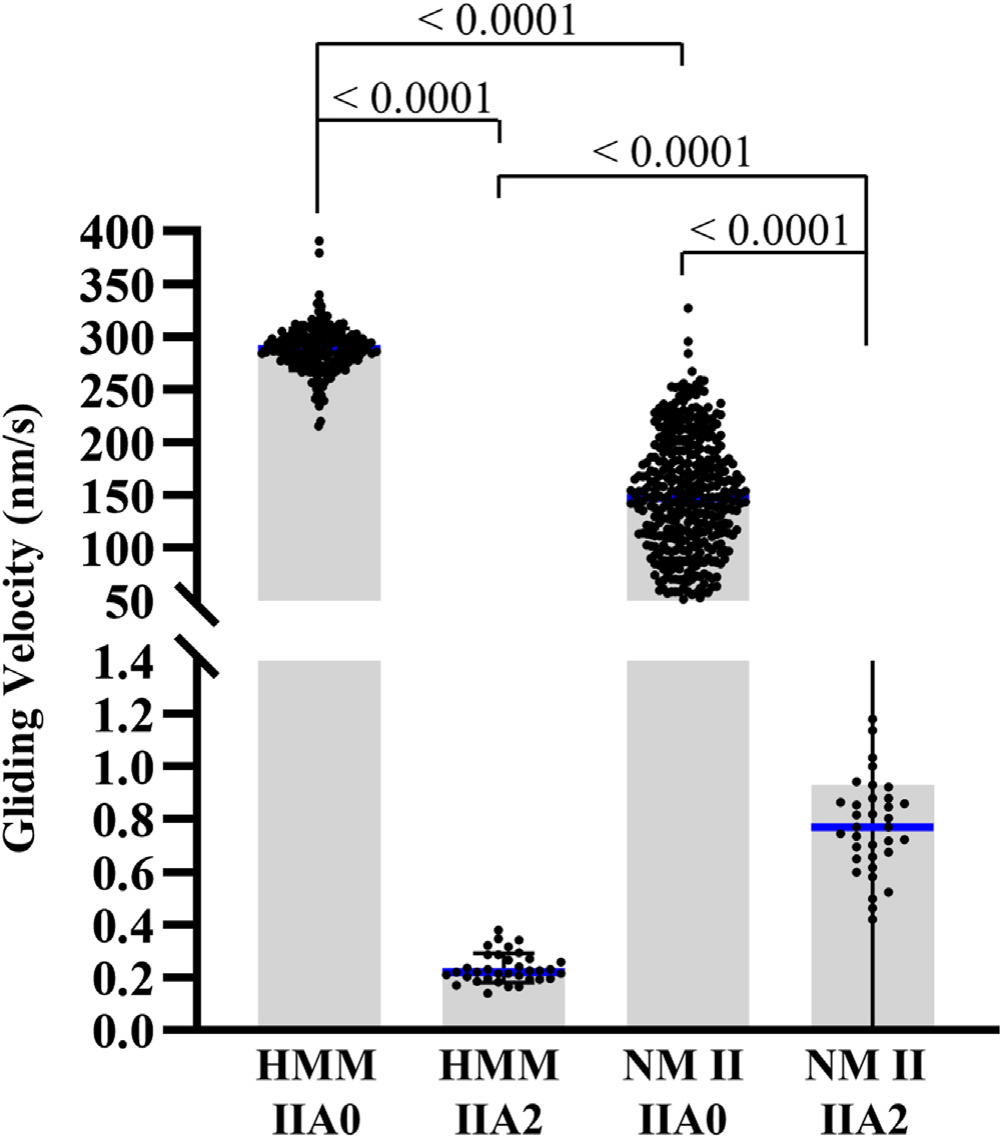
**A2 insert containing isoform do not show the actin-gliding velocity.***In-vitro* motility assay with HMM IIA0, -IIA2, NM IIA0, and -IIA2 in the presence of MLCK. Note that HMM IIA2 or NM IIA2 shows no gliding of actin filaments (Movies S2 and S4). Data are shown as mean ± SD from n = 277 (HMM IIA0), 35 (HMM IIA2), 400 (NM IIA0), and 35 (NM IIA2) actin filaments from three biological replicates. *Blue horizontal line* denotes the median value. Data were analyzed using the one-way ANOVA test followed by pairwise student *t* test. *p* > 0.05 was considered to be nonsignificant. HMM, heavy meromyosin; MLCK, myosin light chain kinase; NM, nonmuscle myosin.

**Figure 4:**
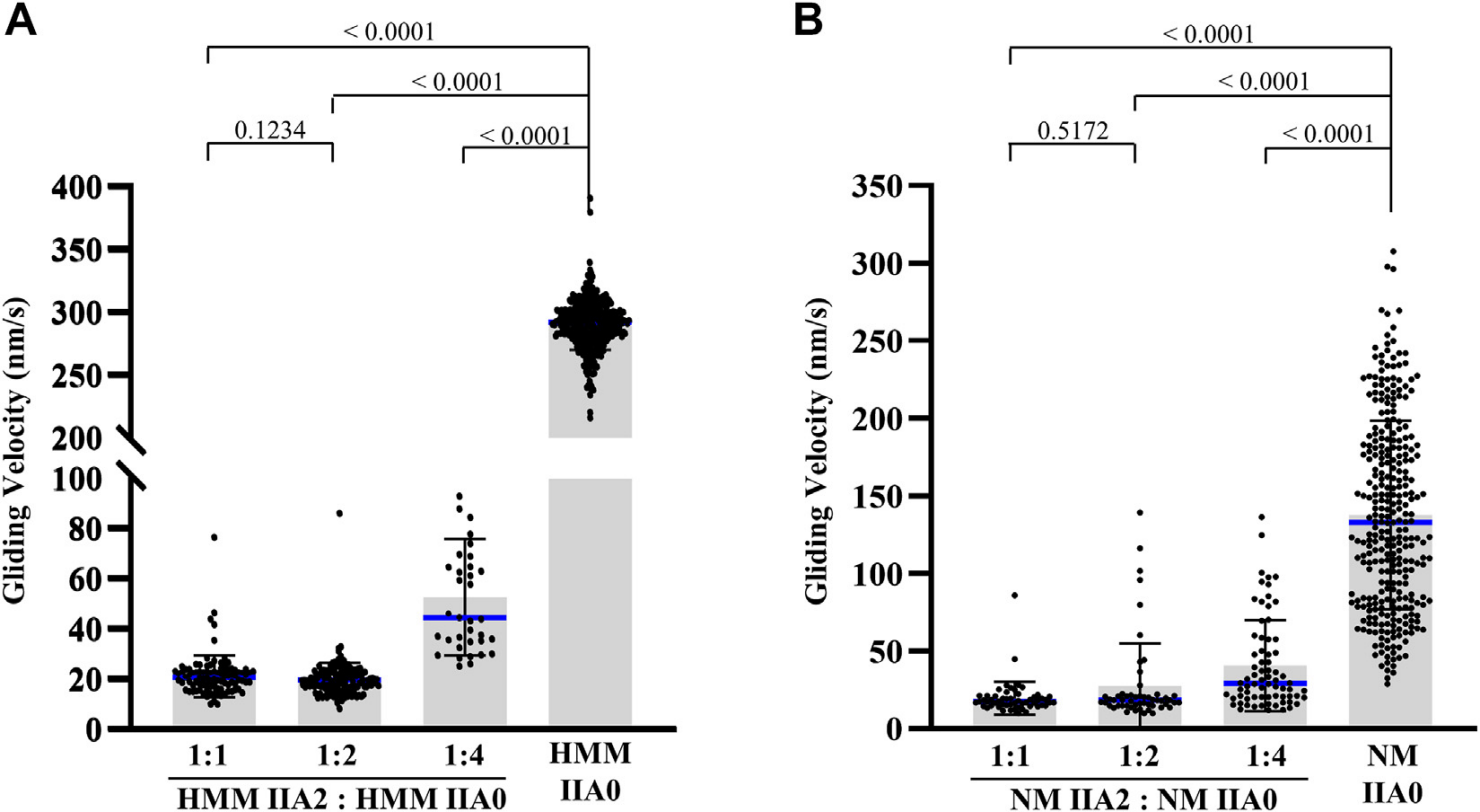
**NM IIA2 reduces the actin-gliding velocity of NM IIA.***A* and *B*, *in vitro* motility assay with HMM IIA (*A*) and NM IIA (*B*), in which a fixed amount of inserted isoform (HMM IIA2 or NM IIA2) was mixed with varying amount of their corresponding noninserted isoform (HMM IIA0 or NM IIA0) at 1:1, 1:2, or 1:4 ratios, as indicated, in the presence of MLCK. *In vitro* motility of noninserted isoforms HMM IIA0 and NM IIA0 only were considered as positive controls for *A* and *B*, respectively. Data are shown as mean ± SD from n = 405 (HMM IIA0), 95 (1:1), 167 (1:2), 36 (1:4), 306 (NM IIA0), 55 (1:1), 54 (1:2), 72 (1:4) actin filaments from three biological replicates. *Blue horizontal line* denotes the median value. Data were analyzed using the one-way ANOVA test followed by pairwise student *t* test. *p* > 0.05 was considered to be nonsignificant. HMM, heavy meromyosin; MLCK, myosin light chain kinase; NM, nonmuscle myosin.

Next, we measured the effect of A2 insert on actin gliding activity of HMM IIA2 and full-length NM IIA2. Both the full-length and HMM IIA molecules were tethered to nitrocellulose-coated coverslips. We observed the movements of the fluorescent labeled-actin filaments along the bed of NM IIAs using total internal reflection fluorescence microscopy. We noticed that phosphorylated HMM IIA0 exhibited a higher mean gliding velocity (274 ± 20 nm/s) of actin filaments than phosphorylated full-length NM IIA0 (147 ± 57.53 nm/s) (Fig. 3, Movies S1 and S2). These values were comparable to previous reports (11). On the contrary, phosphorylated HMM IIA2 and NM IIA2 did not appear to move actin filaments in the assay. The measured velocity was 0.24 ± 0.06 nm/s for HMM IIA2 and 0.93 ± 0.94 nm/s for NM IIA2 (Fig. 3, Movies S3 and S4), demonstrating that insertion of 21 residues near the actin-binding region may significantly reduce or abolish NM II’s ability to move the actin filaments *in vitro*. Notably, the insertion did not affect the ability of HMM IIA2 and NM IIA2 to tether actin filaments to the coverslip surface as the actin filaments remained bound even after the flow chamber was washed with motility buffer containing ATP. This observation prompted us to study the robust effect of A2 in HMM IIA2 and NM IIA2 on modulating the actin gliding activity of HMM IIA0 and NM IIA0, respectively. We mixed HMM IIA2 with HMM IIA0 at different ratios and looked for the movement of actin filaments. Figure 4, *A* and *B* shows that the presence of only 20% A2-bearing myosin in the mixture significantly reduced actin gliding velocity of HMM and full-length proteins to the following (52.8 ± 23.29 nm/s for HMM and 40.64 ± 29.33 nm/s for full-length). At a proportion of 50% A2, further reduction of the velocity to 21.4 ± 8.35 nm/s (Movie S5) and 19.58 ± 10.68 nm/s (Movie S6) was seen for HMM and full-length proteins, respectively. Altogether these data suggest that the presence of A2 inserted isoform decreases overall NM IIA activity.

### NM IIA2 can form bipolar filaments and colocalize with NM IIA0

**Figure 5:**
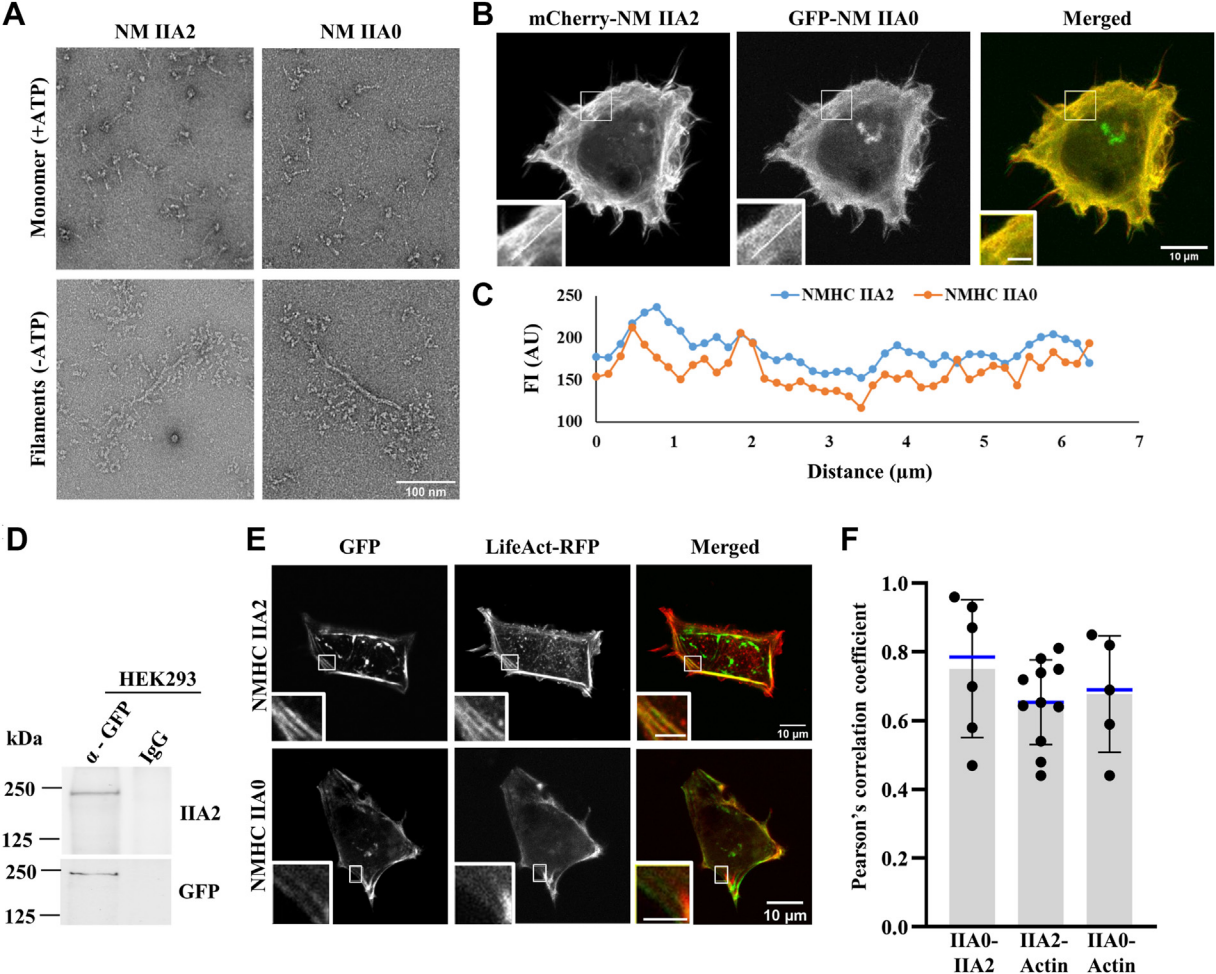
**A2 insert containing isoform form bipolar filament and colocalize with actin filaments.***A*, negative-stain electron microscopy images of full-length GFP-NMHC IIA2 (*left*) and GFP-NMHC IIA0 (*right*) in the presence or absence of ATP. Note that NM IIA2 has similar appearances as NM IIA0. *B*, confocal microscopy images of HEK293 cells coexpressing GFP-NMHC IIA0 and mCherry-NMHC IIA2. *Insets* are the magnified images of the boxes as indicated in the panels. *C*, line-profile of fluorescence intensity (AU) of GFP-NMHC IIA0 and mCherry-NMHC IIA2 across the line indicated in the magnified image in (*B*). *D*, co-immunoprecipitation (Co-IP) of NMHC IIA2 with NMHC IIA0. Cell lysates of HEK293 cells coexpressing GFP-NMHC IIA0 and mCherry-NMHC IIA2 were subjected to IP with α-GFP antibody. The pellets were probed with A2 antibody. Rabbit IgG was used as negative control for IP. GFP antibody was used for input control. *E*, confocal microscopy of HEK293 cells coexpressing Lifeact-RFP and GFP tagged NMHC IIA2 (*upper panel*) or -IIA0 (*lower panel*). Colocalization of actin with exogenouos NMHC IIA2 and NMHC IIA0 is shown in the magnified images, *bottom left corner* of the panels. *F*, quantification of the Pearson’s correlation coefficients shown in *B* and *E*. n = 6 (NM IIA0 and NM IIA2), 5 (NM IIA0 and actin), 11 (NM IIA2 and actin) cells from three independent experiments. Data are represented as mean ± SD. The scale bar represents 2 μm (*inset* magnified images). NM, nonmuscle myosin; NMHC, nonmuscle myosin heavy chain; RFP, red fluorescent proteins.

As the data described above indicate that NM IIA2 has significantly reduced activity (>10-fold reduction), we were interested to check another biological activity of this isoform of NM IIA2. To assess whether A2 insertion can modulate the filamentous property, we carried out negative-stain electron microscopy with purified NM IIA2 and NM IIA0. Figure 5*A* shows that unphosphorylated NM IIA2 can form bipolar filaments in the absence of ATP and remain in the compact and monomeric form in the presence of ATP, a finding similar to a previous report with NM IIA0 (11). To check whether A2 insertion can allow the formation of heterofilaments with NM IIA0, we carried out confocal microscopy and pull-down analyses with HEK293 cells coexpressing GFP-NMHC IIA0 and mCherry-NMHC IIA2. We considered nonneuronal cell line, HEK293 cell, in which NMHC IIA2 expression was not detectable. Figure 5, *B* and *C* shows mCherry-NM IIA2 can colocalize with GFP-NM IIA0 (Pearson’s correlation coefficient, 0.75 ± 0.20). Next, we immunoprecipitated GFP-NMHC IIA0 with GFP antibody and the pellet was probed with A2 insert–specific antibody. We found that mCherry-NMHC IIA2 was coimmunoprecipitated with GFP NMHC IIA0 (Fig. 5*D*), suggesting that NMHC IIA2 can make filaments with NMHC IIA0. NM IIA2 can also colocalize with actin (Pearson’s correlation coefficient, 0.65 ± 0.12, Fig. 5, *E* and *F*). To assess the dynamic behavior of the GFP-NM IIA2 proteins in cells, we performed fluorescence recovery after photobleaching analysis of GFP-NMHC IIA2 and -IIA0. The rate of recovery of the fluorescence intensity by NM IIA2-GFP at the region of interests after photobleaching was almost to the same extent as with NM IIA0-GFP (Fig. S3, *A*–*C*). Altogether these data suggest that NM IIA2 can form *de novo* bipolar filaments, bind to actin filaments and form heterofilaments with NM IIA0 in the cells.

### The expression of NM IIA2 varies with the circadian rhythm

**Figure 6:**
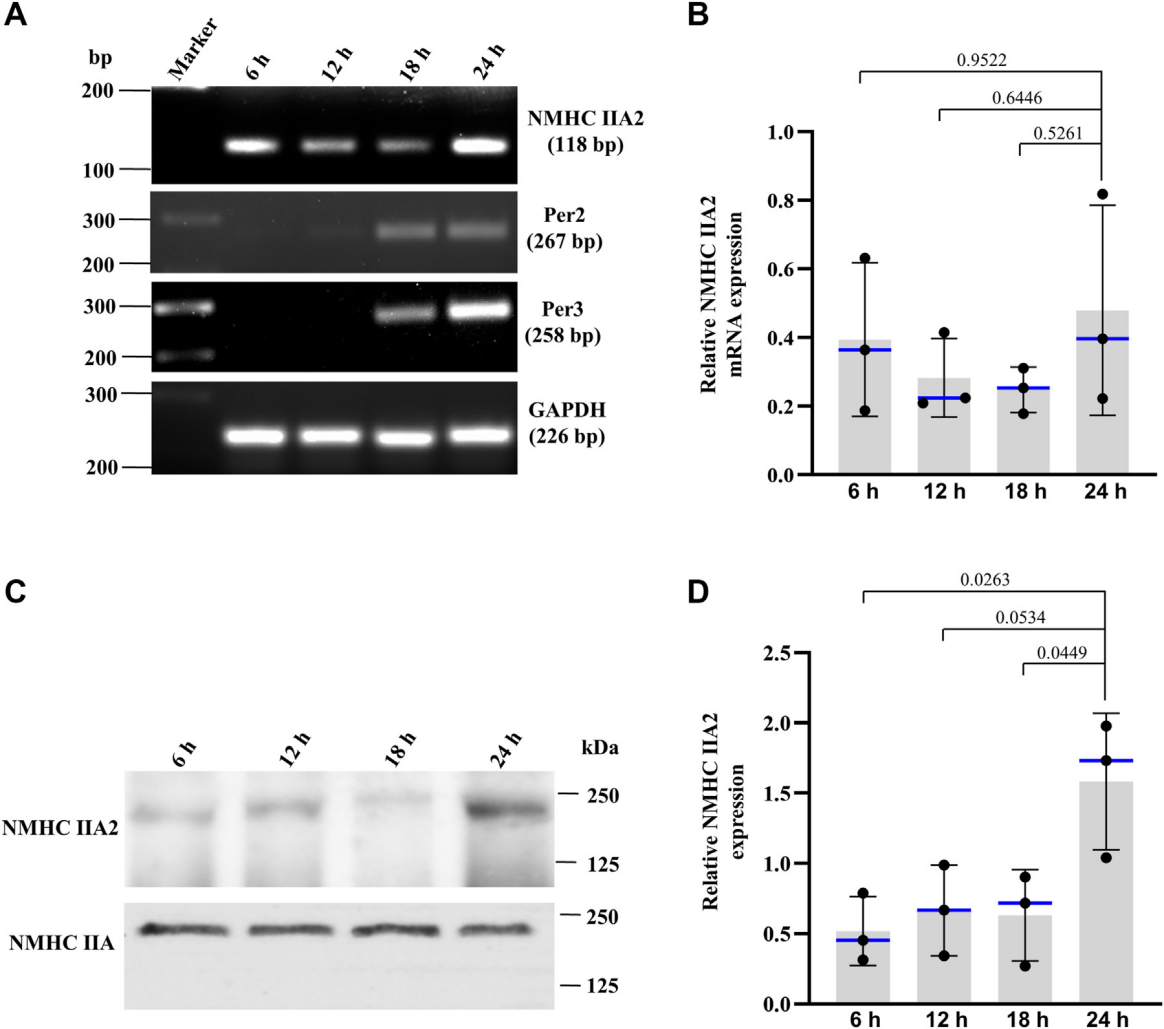
**Variation of NM IIA2 expression with circadian rhythm.***A*, RT-PCR analysis of NMHC IIA2 mRNA during the circadian cycle. *B*, quantification of band intensities of *A*. GAPDH was used as loading control. Relative band intensities were calculated by the following equation; (intensity of A2 band/intensity of GAPDH) at each time point. Per2 and Per3 were used as circadian markers. *C*, immunoblot analysis of NMHC IIA2 in mouse brain at different time points, as indicated during circadian cycle. The endogenous NMHC IIA was immunoprecipitated with an antibody specific to the C-terminal region of NMHC IIA (IIA Ab). The immunoprecipitates were probed with an antibody specific to A2 insert. IIA Ab was used for input control to normalize NMHC IIA2 levels. *D*, quantification of band intensities of *C*. Relative band intensities were calculated as (intensity of A2 band/intensity of input IIA band) at each time point. The experiment was repeated three times from three biological replicates for each time point. Data were analyzed by paired ANOVA followed by Tukey test. Data are represented as mean ± SD. *Blue horizontal lines* denote the median value. *p* > 0.05 was considered to be nonsignificant. The time intervals 6 h, 12 h, 18 h, and 24 h represent 06:00, 12:00, 18:00, and 24:00, respectively, in the 24-h clock format. NMHC, nonmuscle myosin heavy chain; RT-PCR, reverse transcription.

Brain-specific expression of inactive NM IIA2 prompted us to investigate its expression during the circadian cycle to assess the importance of NM IIA2 in brain function. We observed a change in the expression of NMHC IIA2 during circadian rhythm. The expression of NMHC IIA2 both at mRNA (Fig. 6, *A* and *B*) and protein level (Fig. 6, *C* and *D*) was increased to maximum level at 24 h. The variation of the NMHC IIA2 expression in the circadian cycle suggests its possible involvement in brain function.

## Discussion

In this study, we discover an alternatively spliced isoform of NM IIA, NM IIA2 in brain tissue. This isoform is an enzymatically inactive motor molecule and when mixed on the surface with the unspliced NM IIA inhibits the motor activity of the latter.

Our finding on alternative splicing at loop 2 region of NMHC IIA mRNA suggests that splicing event at this position is conserved among NM II family members, though the splicing inserts are different in size. The size of splicing insert at loop 2 of NMHC IIA and IIB mRNAs is 21aa whereas that of IIC mRNA is 33 aa. The loop 2 inserted isoforms of both NM IIB (B2) and IIC (C2) show neuronal tissue specificity and significant differences in their enzymatic and motile properties compared to the noninserted isoforms. Kim *et al.* (16) reported that NM IIB2 exhibited less than 7% *V*_max_ of NM IIB0 in the actin-activated Mg^2+^-ATPase activity and almost no movement in the *in-vitro* motility assay. On the other hand, NM IIC2 does not require light chain phosphorylation to demonstrate its maximum motor activities (9). Our present study on NM IIA2 shows that the insertion at an analogous region of other NM II paralogs brings further diversity to the biochemical properties of the NM II family members. While NM IIB and NM IIC are also alternatively spliced at loop 1, we do not see any evidence of such a splice in NM IIA. We cannot rule out the possibility of very low abundance of NM IIA1 expression or a cell specific expression of NM IIA1 in the tissue, which is limited in our detection method.

RLC phosphorylation does not dramatically activate the enzymatic or motile activities of NM IIA2. There is precedent for this behavior in NM IIB where the splicing at the same loop generates a similarly inactive or less active isoform (16). Myosin 18A has been shown to also be enzymatically inactive, although in this case it is clear that its active site has evolved such that some of the amino acid residues known to be responsible for the binding and hydrolysis of ATP have been lost (20). While myosin 18A does not form filaments by itself, it does coassemble with filaments of NM II (21). Despite the lack of a robust actin-activated activity, NM IIA2 does retain the ability to interact with actin as evidence by its ability to tether actin to the coverslip in the *in vitro* motility assay. It acts as a negative regulator of the *in vitro* motility of phosphorylated NM IIA when the two proteins are mixed in various ratios on the coverslip.

Inside the cell the alternatively spliced and unspliced isoforms appear to colocalize and are likely to form cofilaments due to filament forming tail regions being identical. Despite this, the expression levels of NM IIA2 are significantly lower than those of the unspliced isoform (15% and 5% in mouse brain and muscle, respectively) and this must be considered when assessing any change to mechanical output induced by the presence of the spliced isoform. Nonetheless, the presence of NM IIA2 in a filament would downregulate its mechanical output. It has been shown, for example that the velocity of movement of cofilaments of phosphorylated NM IIA and NM IIB are largely determined by the velocity of the slower NM IIB (22).

We do not believe that the reason for the low ATPase activity of the phosphorylated NM IIA2 is due to it being locked into the interacting head motif present in the autoinhibited conformation. The high-resolution structure of the autoinhibited conformation (10S) of smooth muscle myosin published by Heissler *et al.* (23) was able to visualize the position of the phosphorylated serine-19 for the first time. This study suggested that the presence of the phosphoryl group on this residue would present severe steric clashes between the residues that were stabilizing the complex in this area. Rather, we believe that the residues inserted into loop 2 are directly interfering with the mechanism of actin-activated phosphate release. It is known that substitutions of loop 2 sequences from other myosins with that of the native loop 2 in *Dictyostelium* myosin affect actin-activated Mg^2+^-ATPase rates (24). Similarly, altering the charge and/or length of this loop in the same myosin resulted in varying actin affinity and maximal Mg^2+^-ATPase rates (25). Thus, there is precedent for the effect of this loop on the enzymatic activity of myosins. Having the similar low motor activity of NM IIA2, we can hypothesize that NM IIA2 may have roles in cellular processes that depend more on the structural (filament-forming and actin anchoring) properties than the motor activities. NM IIB2 has been shown to be involved in cerebellar development, particularly with respect to Purkinje cell localization and maturation and controlling the number of dendritic spines and branches associated with Purkinje cells (14). Although we confirmed the brain-specific presence of a novel alternatively spliced isoform NM IIA2, we were not able to detect NMHC IIA2 mRNA in neuronal cell lines such as Neuro 2A, SH-SY5Y, PC-12, suggesting that NM IIA2 is present in very limited abundance or in some specific cell types in the brain, which have not been tested yet. Further investigation on such a low abundance protein, NM IIA2, in brain development or in circadian rhythm is warranted.

Alternative splicing is an important posttranscriptional process which is highly prevalent in the brain tissue (26, 27, 28). Mammalian alternatively spliced isoforms contribute to various neuronal processes, from neuronal development to synaptosis, circadian rhythm maintenance, and others (29, 30). The dysregulation of alternative splicing in brain is known to cause several diseases, highlighting its importance (31, 32, 33). Actomyosin-based contractility participates in various cellular processes, such as neurite outgrowth and growth cone turning, neurite adhesion to the substratum and migration in neuronal cells (34, 35, 36, 37, 38). Actomyosin-based contractility depends on the composition of NM II bipolar filaments and NM II paralogs form heterofilaments that could contain various alternatively spliced isoforms of the HCs (NMHC IIA, IIB, and IIC). In fact, the mammalian RLC gene also exhibit alternatively spliced isoforms (39). The overall activity of the actomyosin complex also depends on the configuration of the filament (40). Therefore, knowing the precise composition and orientation of NM IIA proteins in NM IIA filaments may open up a window to study the functional diversity of NM IIA isoforms in neuronal cells in both disease states and during normal function.

## Experimental procedures

### Cell lines and mice

All Balb/c mice were maintained according to the guidelines approved by the Institutional Animal Ethics Committee of Indian Association for the Cultivation of Science, Kolkata. A 12 h light on/off cycle was maintained at animal room. The tissues from adult Balb/c mice were collected and stored at −80 °C for downstream applications. For the circadian cycle study, brain tissues of Balb/c mice (of 6 weeks of age) were collected at different time points at 6 h intervals in a 24-h clock format, and stored at −80 °C. Human embryonic kidney cell line, HEK293, was procured from *American Type Culture Collection* and maintained following their guidelines. Briefly, cells were cultured in complete Dulbecco's modified Eagle's medium media (with 10% (v/v) fetal bovine serum and 1% (v/v) penicillin–streptomycin) at 37 °C in a humidified incubator in the presence of 5% CO_2_.

### Reverse transcription-PCR analysis

Total RNA from various tissues including the kidney, lung, liver, heart, spleen, skeletal muscle, and brain were isolated from Balb/c mice by using TRIzol method (Thermo Fisher Scientific). The human total RNA from the spinal cord, cerebellum, cerebral cortex, and skeletal muscle were procured from Takara. One microgram of isolated total RNA was treated with DNase I enzyme kit (Sigma-Aldrich) and reverse transcribed using random hexamers, and high capacity complementary DNA (cDNA) reverse transcription kit (Sigma-Aldrich). The cDNA was amplified using a primer set flanking A2 inserted regions and Taq DNA polymerase kit (Sigma-Aldrich). The primer sets were as follows. For human: forward primer, 5′-GGTGATGCAGGAGCAGGGCAC-3′, reverse primer, 5′-CCCGGGCAGTGCGGTCTCCG-3′; and for mouse: forward primer, 5′-CCAGAAGCCCAAGCAACTGAA-3′, reverse primer, 5′-AGGCACCAGGTAGTGCTGTCT-3′. The PCR program included four cycles of denaturation at 95 °C for 1 min, annealing at 62 °C for 1 min, extension at 72 °C for 30 s, and then 31 cycles of denaturation at 95 °C for 1 min, annealing at 58 °C for 1 min, extension at 72 °C for 30 s. To confirm no genomic DNA contamination in the total RNA isolations, sample from skeletal muscle and brain were subjected to cDNA synthesis without reverse transcriptase before the PCR. The PCR products were run on a 1.8% agarose gel followed by EtBr staining. Primers for detecting Per2, Per3, and GAPDH, and A1 and A2 exons were enlisted in Table S2. *In silico* RNA expression was checked in 34 different human tissues from a total of 160 studies from Gene Expression Omnibus (41). The expression profile was enlisted in Fig. S1*B*.

### Sequence analysis of A2 insert and generation of antibody

Reverse transcription-PCR products containing A2 exon from both the mouse brain and human cerebellum were eluted and sequenced. A2 exon with 63 nucleotides were aligned using Clustal Omega. The corresponding amino acid sequence for A2 insert was derived using ExPASy bioinformatics tool. The A2 insert sequence was compared with B2 and C2 amino acid sequence using Clustal Omega for phylogenetic tree analysis. An affinity purified polyclonal antibody against the entire 21 amino acid sequence (EMQSTQRAYFYRRFPILHLES) of the mouse A2 insert were generated in rabbit. The specificity of A2 antibody was checked with mouse tissue extract, exogenous human GFP-NMHC IIA0 and -IIA2 using different dilution. The antibody shows reactivity with both mouse and human A2.

### Immunoprecipitation and immunoblot

Tissue extracts and cell extracts were prepared using a buffer containing 4 mM EDTA, 10 mM MgCl_2_, 1 mM DTT, 0.5 mM PMSF, 5 mM ATP, and protease inhibitor for SDS-PAGE. The tissue extracts or cell extracts for immunoprecipitation were prepared in a buffer containing 10 mM EDTA, 150 mM NaCl, 5 mM ATP, 10 mM MgCl_2_, 1 mM PMSF, 10 μl/ml of protease inhibitor cocktail (P8340, Sigma-Aldrich) and phosphatase inhibitor (P5726, Sigma-Aldrich) and 1 mM DTT (42). The same buffer, lacking DTT, was used for studying heterofilaments. The cell debris was eliminated by centrifugation at 12,000 rpm 4 °C, for 15 min prior to immunoprecipitation. One milligram of total protein was incubated with 2 μg of NMHC IIA antibody (M8064, Sigma-Aldrich), GFP antibody (G1544, Sigma-Aldrich), or IgG (AB002, Biobharti) followed by protein G agarose beads (Sc2002, Santa Cruz Biotechnology) overnight. The tissue and cell extract or pellets were resolved in a 4 to 8% SDS-PAGE, and transferred to a 0.45 mm polyvinylidene fluoride membrane. The blot was blocked with 5% nonfat milk and 0.1% Tween 20 in PBS. The blot was probed with antibodies specific to A2 insert (1:1000), NMHC IIA (M8064, Sigma-Aldrich, 1:5000), Actin (AC004, Abclonal, 1:10,000), or GFP (G1544, Sigma-Aldrich, 1:5000) at 4 °C overnight. Then the blot was incubated with horseradish peroxidase-conjugated mouse or rabbit secondary antibodies for 2 h at room temperature (RT). The blot was incubated with SuperSignal West Femto Maximum Sensitivity Substrate (Thermo Fisher Scientific), and the chemiluminescence signal was captured using a ChemiDoc imaging system (Bio-Rad).

### Preparation of HMM and full-length constructs

Human HMMHC IIA0 (1–4011 nt) tagged with a FLAG sequence was generated from the human full-length NMHC IIA0-GFP construct using primers: forward primer, 5′-ATCGGGCGCGGATCCCGGTCCGAAGCGCGCGGAATTCAAAGGATCTGCAGAATTCGCCCTTATGGCACAGCAAGCTGCCGAT-3′, and reverse primer, 5′-TGCAGGCTCTAGACTACTTATCATCGTCGTCCTTGTAGTCCTCGTCCTCCACCTGCTT-3′, and transferred in pFastBac1 vector at the EcoRI and XbaI sites. The HMMHC IIA0-FLAG was used as a template for the insertion of A2 sequence (63 nt) using the Q5 site-directed mutagenesis kit (New England Biolab). The primers were designed as forward primer, 5′-TCATCGCTTTCCTATTCTCCACCTAGAACCAGTGGACCGCATCAT-3′, and reverse primer, 5′-TAGAAATAGGCTCTCTGAGTGCTCTGCATCTCATCCTTCCACAGCT-3′. The PCR program included initial four cycles of denaturation at 98 °C for 10 s, annealing at 63 °C for 30 s, and extension at 72 °C for 4 min and 30 s followed by 34 cycles of denaturation at 98 °C for 10 s, annealing at 60 °C for 30 s, extension at 70 °C for 4 min and 30 s. Inclusion of A2 sequence in HMMHC IIA0-FLAG was checked by sequencing. HMMHC IIA0-FLAG. Similarly, the full-length inserted NMHC IIA2-GFP and NMHC IIA2-mCherry construct were prepared using the NMHC IIA0-GFP and NMHC IIA0-mCherry as templates, respectively. Insertion of A2 in HMMHC IIA0-FLAG, NMHC IIA0-GFP and NMHC IIA0-mCherry was further confirmed by sequencing. The GenBank accession number for human NMHC IIA2 is OQ689691.

### HMM and full-length myosin purification

The A2 inserted and noninserted HMMHC IIAs and full length NMHC IIAs were expressed in the baculovirus/Sf9 insect culture system along with the two light chains following previously published protocols (11). Briefly, virus containing HC and two light chains were coinfected into 2.5 to 3∗10^9^ Sf9 cells. After 72 h of growth, the infected Sf9 cells were harvested by sedimentation. The pellets were then extracted with an extraction buffer containing 10 mM Mops (pH 7.3), 200 mM NaCl, 10 mM MgCl_2_, 1 mM EGTA, 3 mM NaN_3_, 0.1 mM PMSF, 1 mM DTT, 5 μg/ml leupeptin, and proteinase inhibitor cocktail following homogenization with a Teflon-glass homogenizer. The cell debris was removed by centrifugation in the presence of 1 mM ATP. The proteins were affinity-purified using FLAG affinity chromatography and subjected to dialysis overnight in a dialysis buffer (25 mM NaCl, 10 mM MgCl_2_, 10 mM Mops, 0.1 mM EGTA, and 1 mM DTT, pH 7 for full-length myosin, and 1 mM DTT, 100 mM NaCl for HMM myosin). The concentrations of the proteins were measured by recording absorbance at 280 nm. RLCs of 300 μg purified proteins were subjected to phosphorylation with 0.2 mM CaCl_2_, 3.5 μg/ml MLCK, 0.2 μM calmodulin, 2 μM MgCl_2_, and 1 μM ATP at RT (25 °C) for 30 min (11).

### Denaturing urea gel electrophoresis

Thirty-five micrograms of each phosphorylated and unphosphorylated NM IIAs was precipitated using cold acetone and dissolved in a 6 μl buffer containing 8 M ultrapure urea, 33 mM Tris–glycine (pH 8.6), 0.17 mM EDTA, 10 mM DTT, and 5 mg bromophenol blue. Finally, the samples were subjected to electrophoresis on a 10% Tris–glycine gel at 30 mA for 45 min in a buffer containing 58 mM Tris base, 0.4 M glycine, and 0.05% sodium azide (43).

### Mg^2+^-ATPase activity measurement

The steady-state rate of Actin-activated Mg^2+^-ATPase activities of the spliced isoforms of HMM IIA (with and without regulatory light chains phosphorylation) was measured by an NADH-coupled assay in a spectrophotometer as previously reported (11). Briefly, the assay conditions were 10 mM Mops (pH 7), 2 mM MgCl_2_, 0.15 mM EGTA, 40 units/ml l-lactic dehydrogenase, 200 units/ml pyruvate kinase, 1 mM phosphoenolpyruvate, 0.2 mM NADH, 1 mM ATP, and various concentrations of actin (0–30 μM). The absorbance of NADH was recorded at 340 nm over a time of 30 min at 25 °C. The data were corrected for the background ATPase activity of actin. The kinetic constant *V*_max_ for each assay was measured by fitting the data into the Lineweaver–Burk equation.

### *In-vitro* motility assay

The *in vitro* motility assay of both A2 inserted and noninserted isoforms of HMM IIAs and NM IIAs (with regulatory light chains phosphorylation) was carried out as reported (9). Briefly, assays were performed at 30 °C in a buffer containing 50 mM NaCl, 20 mM Mops (pH 7.4), 5 mM MgCl_2_, 0.1 mM EGTA, 1 mM ATP, 50 mM DTT, 0.7% methylcellulose, 2.5 mg/ml glucose, and 0.1 mg/ml glucose oxidase. NM IIA proteins were introduced at a protein concentration of 0.2 mg/ml into a flow chamber with a nitrocellulose-coated coverslip. The surface was subsequently blocked by 1 mg/ml bovine serum albumin and then incubated for 1 min at RT in a solution containing 5 μM unlabeled F-actin, 1 mM ATP, 0.2 mM CaCl_2_, 1 μM calmodulin, and 4 μg/ml MLCK. After washout, 5 nM F-actin labeled with rhodamine-phalloidin in the assay buffer was applied to the flow chamber. The data were recorded for 2 min, and images were captured at 1 or 5 s intervals using Nikon Eclipse Ti-E total internal reflection fluorescence microscope (CFI 60 Apochromat 100× objective). The gliding speed of actin filaments over the myosin-coated surface was analyzed using the NIS-Elements (Nikon) or Fiji (NIH) software.

### Negative stain electron microscopy

The sample preparation and image acquisition for electron microscopy were carried out as described (11). Briefly, to induce polymerization, NM IIAs were diluted to 100 nM in a buffer containing 10 mM Mops (pH 7.0), 0.1 mM EGTA, 2 mM MgCl_2_ and 150 mM NaCl, and incubated for 30 min on ice. To visualize NM IIAs in the disassembled state, NM IIAs were diluted to 50 nM in the same buffer supplemented with 0.1 mM ATP, and incubated for 30 min followed by crosslinking at RT using 0.1% glutaraldehyde for 1 min. The reaction was quenched by adding Tris–HCl (pH 8) to a final concentration of 100 mM. Proteins were applied to UV treated carbon-coated EM grids, and stained with 1% uranyl acetate. Micrographs were recorded on a JEOL 1200EX microscope using an AMT XR-60 charged-coupled device camera.

### Transfection and super resolution microscopy

For DNA transfection, 1 μg of plasmid DNA/ml was transfected to 2 × 10^5^ HEK293 cells using Lipofectamine 3000 (Invitrogen). Images were captured using Zeiss (LSM 880) super resolution confocal microscope with a 63× oil objective. For the photobleaching experiment, more than one region of interest were selected in HEK293 cells coexpressing GFP tagged NMHC IIA and red fluorescent proteins-tagged Lifeact, and the fluorescent signals were bleached using a 488 nm laser (44). The images were captured at 2 s intervals over 2 to 3 min. The fluorescence intensity in the bleached region was quantified using Fiji software. Photobleaching during imaging was monitored and normalized before drawing the recovery curve. The recovery curve was plotted in Origin software as a relative value of the fluorescence intensities before and after bleaching as 100 and 0, respectively, so that the difference in the protein expression level and the bleaching efficiency between samples were normalized (45). For the colocalization study, Pearson's correlation coefficients were analyzed using Fiji software (Coloc tool).

### Statistical analysis

The data were expressed as a mean ± standard deviation (SD) from more than three biological replicates. Each data point represents mean of three technical replicates. Data sets were tested for normal (Gaussian) distribution *via* the Shapiro–Wilk normality test. The normally distributed data sets were statistically analyzed using one-way ANOVA following the Tukey test, and the nonnormally distributed data sets were analyzed using the Kruskal–Wallis test followed by the pairwise Mann–Whitney U test. The *p* values were provided in the figures, and *p* < 0.05 was considered to be significant.

## Data availability

All data that support the work are available in the article. The complete DNA sequence of human NMHC IIA2 was submitted to GenBank and its accession number is OQ689691.

## Supporting information

This article contains supporting information.

## Conflict of interest

The authors declare that they have no conflict of interest with the contents of this article.
