# Supplementary material for: A brain specific alternatively spliced isoform of nonmuscle myosin IIA lacks its mechanoenzymatic activities
Source: J Biol Chem. 2023 Aug 9;299(9):105143. doi: 10.1016/j.jbc.2023.105143 (PMC10480317; doi:10.1016/j.jbc.2023.105143)
Supplement: Supporting information [file mmc1.pdf]

Supporting Information

A brain specific alternatively spliced isoform of nonmuscle myosin IIA lacks its mechano-enzymatic activities

Samprita Das[1], Ditipriya Mallick[1], Sourav Sarkar[1], Neil Billington[2], James R. Sellers[2*], Siddhartha S. Jana[1*]

[1]School of Biological Sciences, Indian Association for the Cultivation of Science, India

[2]Laboratory of Molecular Physiology, National Heart, Lung and Blood Institute, National Institutes of Health, USA

Short title: Alternatively spliced isoform of non-muscle myosin IIA

*Corresponding authors. JRS at sellersj@nhlbi.nih.gov and SSJ at bcssj@iacs.res.in

Keywords: Nonmuscle myosin II, Alternative splicing, Myosin filament formation, Actin-gliding activity, Enzymatic Mg^{2+}-ATPase activity, Circadian rhythm

Table S1. ATPase activity of HMM IIA isoforms: Basal ATPase activity (s^{-1}) and V_{max} (s^{-1}) of HMM IIA0 and HMM IIA2 in the absence and presence of MLCK. uP, unphosphorylation; P, phosphorylation.

Myosin	Basal ATPase activity (at 0 µM Actin) (s^{-1})	V_{max} (s^{-1})
HMM IIA0-P	0.0561±0.0372	0.3809 ± 0.22
HMM IIA0-uP	0.0012±0.0008	0.0016 ± 0.0008
HMM IIA2-P	0.0045±0.0012	0.0173 ± 0.004
HMM IIA2-uP	0.0021±0.0009	0.0043 ± 0.0008

Table S2: Sequence of the primers used for RT-PCR

Target	Primers	Primer Sequence
Mouse loop1 regions	Forward (P1)	5'-GCACATGTGGCCTCCTCACAC-3'
	Reverse (P2)	5'-ATGTGGAAGGTCCGCTCCTCT-3'
Mouse A2 exon specific	Forward (P3)	5'-AGCACTCAGAGAGCCTATTTCT-3'
	Reverse (P4)	5'-AGGCACCAGGTAGTGCTGTCT-3'
Mouse GAPDH	Forward	5'-GACAACTTTGGCATTGTGGAA-3'
	Reverse	5'-ACACATTGGGGGTAGGAACA-3'
Mouse Per2	Forward	5'-ACCAGCCTCCTTGCTCCTA-3'
	Reverse	5'-ACTGGCTGGTGAGGGACA-3'
Mouse Per3	Forward	5'-CACGTCAGCATCACCTCTTC-3'
	Reverse	5'-GCCAGTATCCGTGGTGCTT-3'

Figure S1: Detection of alternative splicing at loop1 and loop2 of NMHC-IIA. (A) RT-PCR analysis of total RNA isolated from various mouse tissues, as indicated. Bands at 254bp indicates no inclusion of A1 exon. (B) The expression of NMHC IIA2 mRNA in human (reads per million) from Gene Expression Omnibus database. Note that the expression of A2 exon in human was restricted to brain and skeletal muscle tissues.

Figure S2: MLCK phosphorylates RLC of HMM IIA2. A) A representative of glycerol-urea polyacrylamide gel of HMM IIA2 and -IIA0. Bacculovirus expressed HMM IIs were treated with or without MLCK, and run-on glycerol-urea gel followed by silver staining. Note that phosphorylated RLCs (P-RLC) move faster than unphosphorylated RLCs (uP-RLC), as indicated. Experiment was repeated three times.

Figure S3: FRAP analysis of GFP-NMHC IIA2 and -IIA0 in HEK293 cells: A) The representative time lapse images of HEK293 cells expressing GFP-NMHC IIA2 (*upper two panels*) or -IIA0 (*lower two panels*) before and after photobleaching. The region of interests (ROIs), which were photo-bleached, have been depicted with the boxes in the panels. B-C) Quantification of (%) fluorescence recovery (B) and mobile fraction (C) from the FRAP analysis of GFP-NMHC IIA2 or -IIA0 expressing HEK293 cells. n=10 ROIs from 7 cells from three independent experiments. Data were analyzed by Mann-Whitney test, and represented as mean ± SD. Blue horizontal lines denote the median value. FI, fluorescence intensity. Scale bar- 10 μm.

Supporting Movies:

The corresponding purified myosins were tethered to a coverslip and the motility of rhodamine-phalloidin labelled actin filaments were captured under the TIRF microscope. The images were captured at an interval of 2s. All the movies were played at 7fps. Movies are representative of experiments from three separate protein preparations.

Movie 1: Purified HMM IIA0

Movie 2: Purified NM IIA0

Movie 3: Purified HMM IIA2

Movie 4: Purified NM IIA2

Movie 5: HMM IIA0 mixed with HMM IIA2 at a ratio of 1:1

Movie 6: NM IIA0 mixed with NM IIA2 at a ratio of 1:1

A

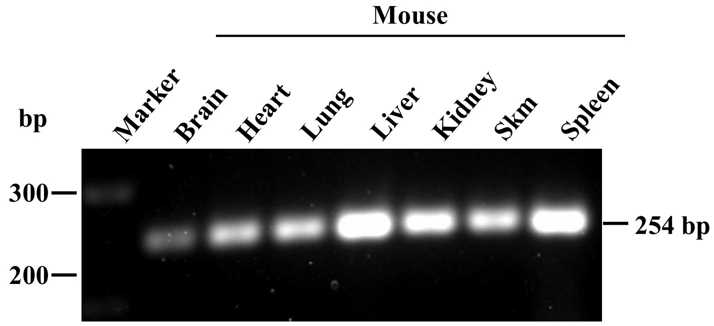
Mouse
bp
Marker
Brain
Heart
Lung
Liver
Kidney
Skm
Spleen
300
200
254 bp

B

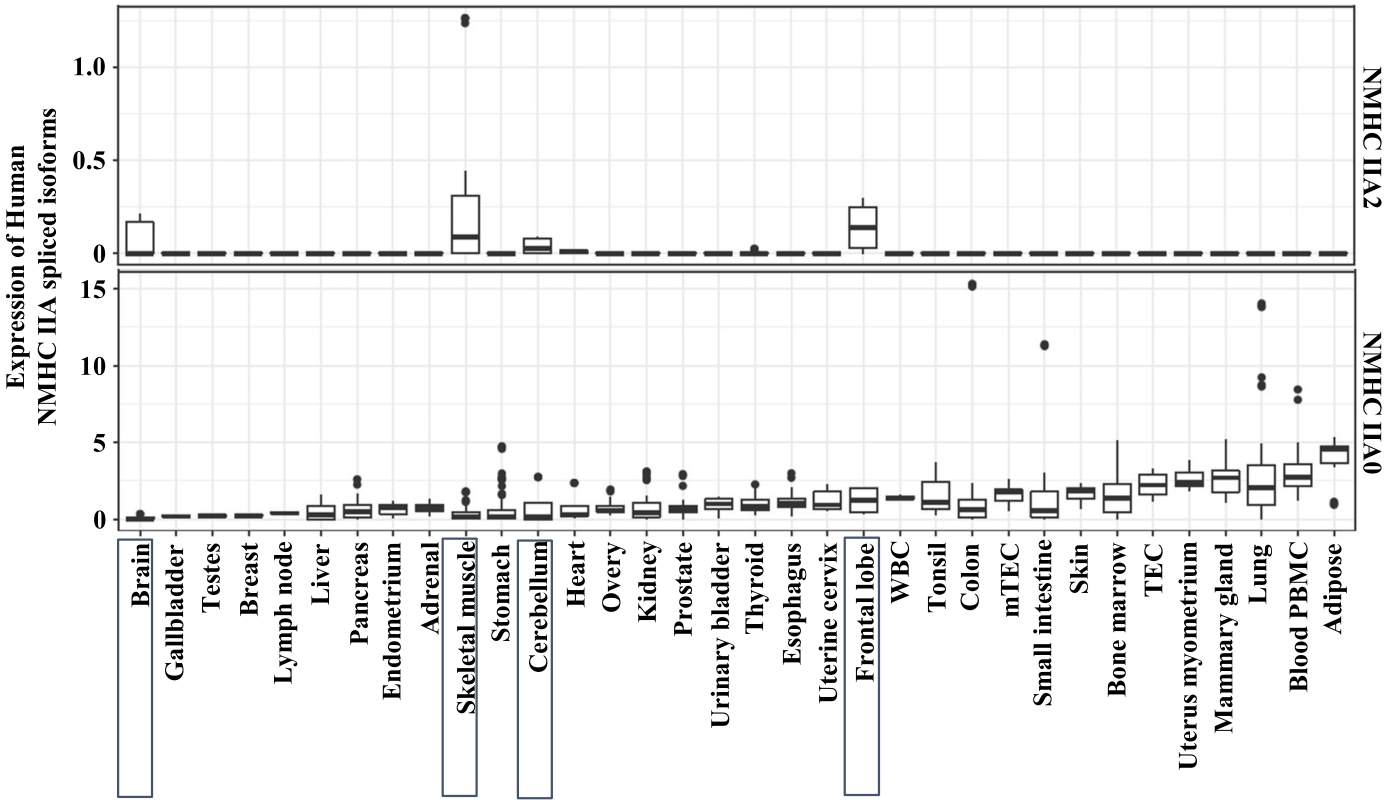
Expression of Human
NMHC IIA spliced isoforms
NMHC IIA2
NMHC IIA0
1.0
0.5
0
15
10
5
0
Brain
Gallbladder
Testes
Breast
Lymph node
Liver
Pancreas
Endometrium
Adrenal
Skeletal muscle
Stomach
Cerebellum
Heart
Overy
Kidney
Prostate
Urinary bladder
Thyroid
Esophagus
Uterine cervix
Frontal lobe
WBC
Tonsil
Colon
mTEC
Small intestine
Skin
Bone marrow
TEC
Uterus myometrium
Mammary gland
Lung
Blood PBMC
Adipose

Figure S1

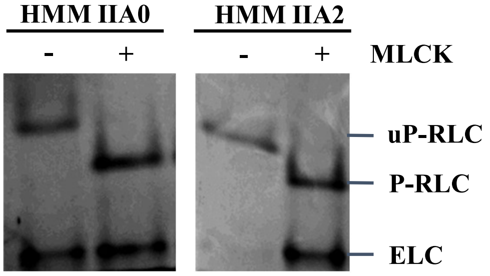

Figure S2

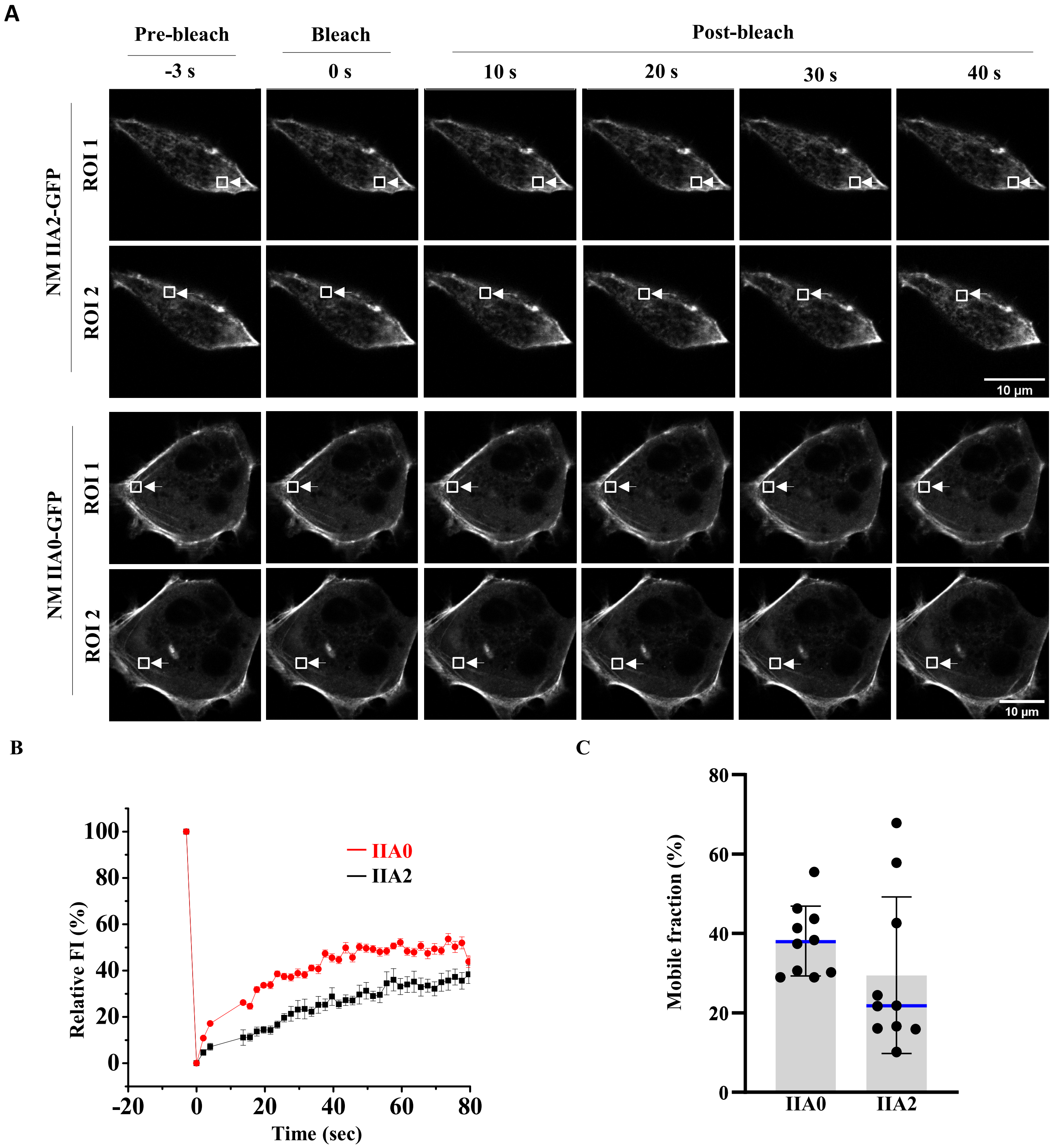

A
Pre-bleach
Bleach
Post-bleach
-3 s
0 s
10 s
20 s
30 s
40 s
NM IIA2-GFP
ROI 1
ROI 2
10 µm
NM IIA0-GFP
ROI 1
ROI 2
10 µm
B
IIA0
IIA2
Relative FI (%)
Time (sec)
C
Mobile fraction (%)
IIA0
IIA2

Figure S3
